# MPP8 Governs the Activity of the LIF/STAT3 Pathway and Plays a Crucial Role in the Differentiation of Mouse Embryonic Stem Cells

**DOI:** 10.3390/cells12162023

**Published:** 2023-08-08

**Authors:** Heyao Zhang, Tenghui Yang, Hao Wu, Wen Yi, Chunhong Dai, Xi Chen, Wensheng Zhang, Ying Ye

**Affiliations:** 1 Cam-Su Genomic Resource Center, Medical College of Soochow University, Suzhou 215123, China; 2 Shenzhen Key Laboratory of Gene Regulation and Systems Biology, School of Life Sciences, Southern University of Science and Technology, Shenzhen 518055, Chinachenx9@sustech.edu.cn (X.C.); 3 Zhejiang Stem and Ageing Research (Z-StAR) Institute, International Campus, Zhejiang University, Haining 314400, China

**Keywords:** mESCs, MPP8, LIF/STAT3 signaling pathway, Nanog, differentiation

## Abstract

Mouse embryonic stem cells (mESCs) possess the remarkable characteristics of unlimited self-renewal and pluripotency, which render them highly valuable for both fundamental research and clinical applications. A comprehensive understanding of the molecular mechanisms underlying mESC function is of the utmost importance. The Human Silence Hub (HUSH) complex, comprising FAM208A, MPP8, and periphilin, constitutes an epigenetic silencing complex involved in suppressing retroviruses and transposons during early embryonic development. However, its precise role in regulating mESC pluripotency and differentiation remains elusive. In this study, we generated homogenous miniIAA7-tagged *Mpp8* mouse ES cell lines. Upon induction of MPP8 protein degradation, we observed the impaired proliferation and reduced colony formation ability of mESCs. Furthermore, this study unveils the involvement of MPP8 in regulating the activity of the LIF/STAT3 signaling pathway and Nanog expression in mESCs. Finally, we provide compelling evidence that degradation of the MPP8 protein impairs the differentiation of mESC.

## 1. Introduction

ESCs, derived from the inner cell mass of blastocysts, possess the remarkable capabilities of indefinite self-renewal and the potential to differentiate into multiple cell types [1,2]. The self-renewal of ESCs is tightly regulated by a complex interplay of transcription factors (TFs), signaling pathways, and microRNAs [3,4,5]. Numerous studies have emphasized the crucial role of epigenetic regulation in maintaining the self-renewal and pluripotency of ESCs [6,7,8]. Epigenetic regulators, including the Polycomb group (PcG) complex, nucleosome remodeling complexes, histone deacetylase (HDAC) complexes such as NuRD (nucleosome remodeling and deacetylase), BAF (BRG1-associated factors) complex, and DNA methyltransferases (DNMTs), have been identified as critical players in ESC self-renewal and pluripotency maintenance [9,10,11,12,13,14].

In 2015, Tchasovnikarova et al. discovered the Human Silencing Hub (HUSH) complex, composed of three subunits: MPP8, FAM208A, and periphilin [15]. Studies have revealed that the HUSH complex is involved in gene silencing through its interaction with H3K9me3 [16,17,18,19,20]. Furthermore, Mpp8-regulated gene silencing has been linked to the regulation of DNA methylation [19,21].

Recent studies have demonstrated that MPP8 participates in promoting or inhibiting the proliferation, invasion, and migration of different types of cancer cells [19,22,23,24,25]. It has also been implicated in silencing retroviral DNA and transposons [17,18,26,27,28,29]. The function of the HUSH complex in ESCs has been reported recently [18,30,31]. Robbez-Masson et al. demonstrated the repression of the HUSH complex on young retrotransposons in naïve mouse ESCs [18]. The HUSH complex recruits NEXT (nuclear exosome targeting) to control the expression of transposable element (TE) RNA in ESCs [30]. Depletion of MPP8 leads to cell cycle arrest and spontaneous differentiation, indicating its essential role in ground-state pluripotency [31]. However, the precise function and molecular mechanism of the HUSH complex in ESC differentiation regulation remain unknown.

The auxin-inducible degron (AID) is a recently discovered technology for rapid protein degradation [32]. This innovative approach has been successfully employed to achieve rapid degradation of endogenous proteins in various cell types, including murine, monkey, and human cells [32,33,34].

In this study, we generated Mpp8^AID^ mouse ES cell lines to investigate the role of MPP8 in the self-renewal and differentiation of ESCs. Our findings indicate that depletion of MPP8 impairs the proliferation and colony formation ability of mESCs. Notably, this study reveals that the degradation of MPP8 leads to increased expression of key pluripotency gene Nanog, as well as increased activity of the LIF/STAT3 pathway. Finally, we demonstrate that the deletion of Mpp8 impairs the differentiation of mESCs.

## 2. Materials and Methods

### 2.1. Cell Culture and Differentiation

ESCs were cultured in a medium comprising DMEM (Procell, Wuhan, China, cat. no. WH0021U211) supplemented with 10% fetal bovine serum (Labtech, Beijing, China, cat. no. FCS-SA/500), 1% L-Glutamine (Gibco, Waltham, MA, USA, cat. no. 25030-081), 1% penicillin-streptomycin (Gibco, Waltham, MA, USA, cat. no. 15140-122), 0.1% β-Mercaptoethanol (Sigma, St. Louis, MO, USA, cat. no. M3148), and 10^3^ U/mL mLIF (GenScript, Nanjing, China, cat. no. Z03077). For differentiation, ESCs were dissociated using 0.05% Trypsin-EDTA (Gibco, Waltham, MA, USA, cat. no. 2072820) and resuspended in ES medium without LIF. Approximately 6~8 × 10^5^ ESCs were then plated in low-attachment Petri dishes and incubated at 37 °C with 5% CO_2_ to induce the formation of embryoid bodies (EBs). The EBs were collected at the indicated time for qPCR analysis.

Mpp8^AID^ cells served as the control throughout the entire study, unless otherwise specified.

### 2.2. Generation of AID-Tagged ESCs and Mpp8-Inducible Overexpression ESCs

To obtain homogeneous AID-tagged Mpp8 ES cells, Rosa26-OsTIR1 ESCs were transfected with the linearized PL-Mpp8-Flag-AID-EGFP vector, the corresponding sgRNA vector, and Cas9-encoding vector using Lipo8000 transfection reagent (Beyotime, Shanghai, China, cat. no. C0533FT). Following transfection, colonies were selected using a combination of 250 μg/mL G418 (Sangon Biotech, Shanghai, China, cat. no. GDJ958) and 1 μg/mL of Puromycin (Sigma, St. Louis, MO, USA, cat. no. P8833). Finally, clones were selected for genotyping.

To generate inducible Mpp8 overexpressing ESCs, the Mpp8 cDNA was amplified and inserted into the pPBH-TREtight-MLC-EGFP vector. The Mpp8 overexpression vector and PBase vector were then transfected and randomly integrated into the genome of the Mpp8AID ESCs. Colonies were selected for genotyping after being cultured in the selection medium containing 100 μg/mL hygromycin (Invitrogen, Waltham, MA, USA, cat. no. 10687010). A culture medium supplemented with 1 μg/mL Doxycycline (Sigma, St. Louis, MO, USA, cat. no. 3219-99-6) was used to induce the overexpression of Mpp8.

### 2.3. Western Blot Analysis

Proteins were separated via SDS-PAGE and transferred onto a PVDF membrane (Sigma, St. Louis, MO, USA, cat. no. IPVH00010). Subsequently, the membrane was blocked with 5% skimmed milk (Fdbio Science, Hangzhou, China, cat. no. FD0080) in TBST buffer at room temperature for 1 h. Following the blocking step, the membrane was incubated with primary antibodies overnight at 4 °C on a shaker, followed by three washes with TBST. Next, the membrane was incubated with secondary antibodies at room temperature for 1 h on a shaker, and then washed three times with TBST. Finally, protein bands were visualized using an enhanced chemiluminescence (ECL) Plus system (Vazyme, Nanjing, China, cat. no. L/N 7E530C1) and scanned with a ChemiScope series Imaging System (Clinx Science Instruments Co., Ltd., Shanghai, China, cat. no. 6300).

Primary antibody: OCT4 (SantaCruz, SantaCruz, California, USA, cat. no. SC-5297, 1:1000), SOX2 (Abcam, Cambridge, UK, cat. no. Ab97959, 1:500), NANOG (Abcam, Cambridge, UK, cat. no. Ab80892, 1:1000), STAT3 (SantaCruz, SantaCruz, California, USA, cat. no. SC-8019, 1:500), p-STAT3 Tyr705 (CST, Leiden, Netherlands, cat. no. 9131s, 1:1000), MPP8 (proteintech, Wuhan, China, cat. no. I6796-I-AP, 1:500). 

### 2.4. RT-PCR Analysis

For the RT-qPCR analysis, total RNA was purified using a FastPure Cell/Tissue Total RNA Isolation Kit V2 (Vazyme, Nanjing, China, cat. no. RC112-01), following the manufacturer’s instructions. The purified RNA was then reverse-transcribed using 5× HiScript II Q RT SuperMix (Vazyme, Nanjing, China, cat. no. R222-01-AB), per the manufacturer’s recommendations.

RT PCR was performed using the 2× ChamQ Universal SYBR qPCR Master Mix (Vazyme, Nanjing, China, cat. no. Q711-02-AA). The mRNA expression levels were normalized using *Gapdh* as a reference gene. The primer sequences for qPCR can be found in Supplementary Table S2.

### 2.5. Alkaline Phosphatase (AP) Staining

A total of 5000 cells were cultured in a gelatin-coated 10cm dish. After 7–10 days, when distinct clones became visible, the ES cells were washed twice with DPBS. Subsequently, the cells were fixed with 4% paraformaldehyde for 2–5 min and stained using the Alkaline Phosphatase Kit (Sigma, St. Louis, MO, USA, cat. no. SCR004) according to the manufacturer’s instructions. The staining reaction was halted by rinsing the cells with PBS three times. The stained cells were then counted and subjected to statistical analysis.

### 2.6. Cell Growth Curve Analysis

ESCs were dissociated using 0.05% trypsin-EDTA and seeded onto a 10 cm dish. The cells were then cultured for 8 days, with cell counting performed every 48 h. The collected cell counting results were subjected to statistical analysis. Subsequently, a growth curve was plotted, representing the cell number on the vertical axis and the cell culture time on the horizontal axis.

### 2.7. RNA-Seq Experiments

Total RNA was extracted using the RNA Isolation Kit (Vazyme, Nanjing, China, cat. no. RC101-01) following the manufacturer’s instructions. The concentration of RNA was determined using a NanoDrop spectrophotometer. RNA-seq experiments were performed as previously described [12].

### 2.8. ChIP-Seq Data Analysis

In this study, public ChIP-seq data from refs. [12,29] were utilized. The ChIP-seq data analysis was conducted following previously described methods [12].

### 2.9. Statistical Analysis

All data are presented as mean ± standard deviation (SD). Statistical significance was determined using a two-tailed Student’s *t*-test. All experiments were independently repeated at least three times. Significance is indicated as * *p* < 0.05, ** *p* < 0.01, *** *p* < 0.001, **** *p* < 0.0001.

## 3. Results

### 3.1. Depletion of MPP8 Impairs the Self-Renewal of mESCs

**Figure 1:**
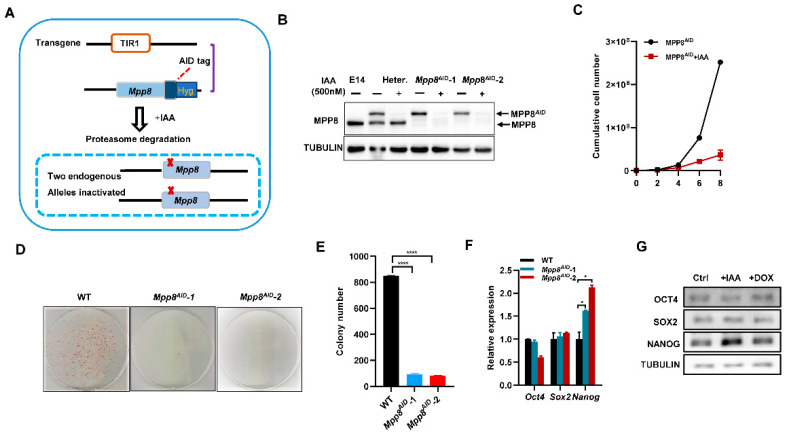
The impact of MPP8 depletion on the self-renewal of ES cells. (**A**) The experimental scheme outlines the generation of Mpp8^AID^ ESCs. Endogenous Mpp8 was inactivated in ESCs using Crispr/Cas9 technology in combination with the Tir1 transgene. Mpp8AID ESCs were generated by transfecting the PB-AID-tagged exogenous Mpp8 vector after the selection process. Further details can be found in the methods section. (**B**) A Western blot analysis was conducted to evaluate MPP8 protein levels in control samples, a heterozygote clone, Mpp8^AID^-1, and Mpp8^AID^-2 clones, both with and without 500 µM IAA treatment. TUBULIN was used as a loading control. The control utilized in this study was E14 wild-type ESCs. (**C**) A cell proliferation assay was performed for Mpp8^AID^ ESCs, with and without 500 µM IAA treatment. (**D**) Representative images depict Mpp8^AID^ ES clones treated with and without 500 µM IAA after AP staining. (**E**) Quantification of the colonies shown in (**D**) revealed a significant difference (**** *p* < 0.0001). (**F**) qPCR analysis measured the transcript levels of core pluripotency genes in WT, Mpp8^AID^-1, and Mpp8^AID^-2 ESCs treated with IAA(* *p* < 0.05). (**G**) Western blot analysis was conducted using the indicated antibodies in control samples, MPP8-depleted ESCs, and Mpp8-overexpressing ESCs.

AID is a rapid protein degradation strategy [32]. To investigate the function of the HUSH complex in ESCs, we generated a cell line called Mpp8^AID^ by introducing a double-strand insertion of AID through homologous recombination (Figure 1A). After genotyping, two clones (Mpp8^AID^-1 and Mpp8^AID^-2) with both alleles of the Mpp8 gene fused with miniAID were obtained. Western blot analysis confirmed the degradation of AID-tagged MPP8 upon treatment with indole-3-acetic acid (IAA) (Figure 1B).

Next, we examined the role of MPP8 in the maintenance of ESCs. The degradation of AID-tagged MPP8 significantly impaired ESC proliferation (Figure 1C). Colony assay experiments revealed a noticeable decrease in colony formation in the absence of MPP8 (Figure 1D,E). Furthermore, the deletion of Mpp8 led to an increase in both the transcript and protein levels of *Nanog* expression, while *Oct4* and *Sox2* levels remained unaffected (Figure 1F,G).

In conclusion, depletion of MPP8 significantly impairs colony formation and inhibits the proliferation of ESCs, albeit with a slight upregulation of Nanog levels.

### 3.2. Degradation of MPP8 Enhances the Activity of the LIF/STAT3 Pathway and Hinders the Transition from Naïve to Primed ESCs

**Figure 2:**
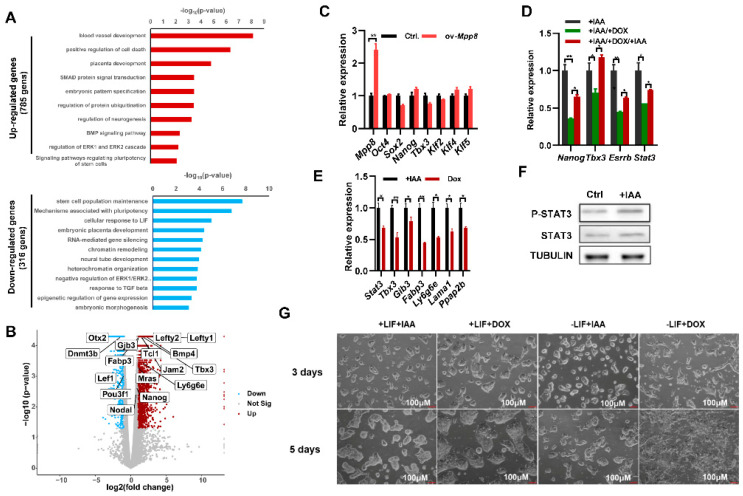
Mpp8’s role in ESC maintenance through Nanog regulation and the LIF/STAT3 pathway. (**A**) GO analysis revealing biological processes associated with differentially expressed genes upon MPP8 depletion in ESCs. (**B**) Volcano plot illustrating differentially expressed genes between WT and MPP8-depleted ESCs. Red dots represent genes upregulated in MPP8-depleted ESCs, while blue dots indicate genes downregulated in WT ESCs. Y-axis represents −log10 P values, and X-axis shows log2 fold change values. Volcano plot generated using GraphPad Prism version 8.2.0. (**C**) qPCR analysis of pluripotency genes in Mpp8-overexpressing ESCs(** *p* < 0.01). (**D**) qPCR analysis of specific genes in ex-Mpp8:Mpp8^AID^ ESCs under different treatment conditions(* *p* < 0.05, ** *p* < 0.01). (**E**) qPCR analysis of LIF/STAT3 target genes in MPP8-depleted and Mpp8-overexpressing ESCs(* *p* < 0.05, ** *p* < 0.01). (**F**) Western blot analysis of STAT3 and p-STAT3 levels in control and MPP8-depleted ESCs upon IAA treatment. (**G**) Morphology of ex-Mpp8:Mpp8^AID^ ESCs after 3 and 5 days of culture under different treatments. Scale bars: 100 μm.

To investigate the impact of Mpp8 on the self-renewal of mESCs, we employed RNA sequencing (RNA-seq) to examine global gene expression changes upon Mpp8 deletion in mESCs. Our analysis revealed 316 significantly downregulated genes and 785 upregulated genes in Mpp8-depleted ESCs compared to control ESCs (Figure 2A; Table S1). Consistent with the impaired colony formation ability (Figure 1D,E), gene ontology (GO) analysis demonstrated the enrichment of apoptosis-associated terms in upregulated genes in Mpp8-depleted ESCs (Figure 2A). The downregulated genes were associated with stem cell population maintenance (Figure 2A), consistent with the impaired proliferation observed in mESCs upon Mpp8 deletion (Figure 1C–E). Notably, RNA-seq analysis revealed the upregulation of typical target genes of the LIF/STAT3 pathway, including Fabp3, Gjb3, Tcl1, Mras, Bmp4, Tbx3, Ly6g6e, Jam2, Lefty1, and Lefty2 (Figure 2B). GO analysis revealed the enrichment of genes associated with the cellular response to LIF in deregulated genes (Figure 2A), indicating the regulation of Mpp8 on the LIF/Stat3 pathway. 

One limitation of the AID system is its basal degradation, where AID-tagged target proteins are degraded before the addition of IAA [34]. To mitigate the effect of basal degradation, we generated ex-Mpp8:Mpp8^AID^ cell lines by transfecting a doxycycline (Dox)-induced Mpp8 overexpression vector into Mpp8^AID^ cells. This allowed us to manipulate Mpp8 expression without the variability associated with clonal effects. Overexpression of Mpp8 in WT ESCs did not affect the expression of major pluripotency genes, including Oct4, Sox2, Nanog, Tbx3, Klf2, Klf4, and Klf5 (Figure 2C). With the ex-Mpp8:Mpp8^AID^ cell lines, we examined the expression of pluripotency genes under three conditions: condition 1 involved IAA-induced degradation of MPP8 for 24 h; condition 2 entailed withdrawal of IAA, while inducing Mpp8 overexpression with the addition of doxycycline for 24 h; condition 3 involved IAA-induced degradation of MPP8, while withdrawing doxycycline for 2 days. Consistent with the repression of Mpp8 on Nanog expression (Figure 1F,G), qPCR analysis demonstrated the highest expression of Nanog in ESCs when MPP8 was degraded upon IAA treatment, while overexpression of Mpp8 led to the lowest Nanog expression. Nanog expression was upregulated again when IAA was added to induce the degradation of MPP8 (Figure 2D). A similar expression pattern was observed for Stat3, and its target genes Tbx3 and Esrrb (Figure 2D). Furthermore, the expression of other LIF/STAT3 target genes, including Gjb3, Fabp3, Ly6g6e, Lama1, and Ppap2b, was significantly higher in MPP8-degraded ESCs compared to ESCs with Mpp8 overexpression (Figure 2E). Consistent with this, Western blot analysis showed increased protein levels of STAT3 and p-STAT3 in ESCs with MPP8 degraded upon treatment with IAA (Figure 2F). Therefore, Mpp8 represses the activity of the LIF/STAT3 pathway in ESCs.

Previous studies have reported that overexpression of Nanog maintains the self-renewal of mESCs in LIF-deficient medium [35,36]. The LIF/STAT3 signaling pathway has been demonstrated to support the propagation of mouse ESCs [37] and the activation of Stat3, and its downstream target genes enables mESCs to maintain self-renewal [38,39]. Temporarily increasing STAT3 activity is sufficient to reprogram human PSCs into naive-like pluripotent cells [40]. Since the degradation of MPP8 increased Nanog expression and the activity of the LIF/STAT3 pathway (Figure 1F,G and Figure 2D–F), we speculated that the deletion of Mpp8 may enhance the maintenance of ESCs. Indeed, Mpp8 overexpression led to obvious differentiation morphology under LIF-deficient culture conditions. In contrast, degradation of the MPP8 protein upon IAA treatment exhibited typical undifferentiated ESC morphology even in the absence of LIF in the medium (Figure 2G). This effect could be attributed to the degradation of MPP8, which subsequently upregulated Nanog expression, thereby maintaining the pluripotent state of ESCs. Notably, RNA-seq analysis showed decreased expression of Otx2, Dnmt3b, Pou3f1, Lef1, and Nodal, which are typical genes of formative and primed ESCs [41,42,43]. Therefore, we conclude that the deletion of Mpp8 may enhance the maintenance of naive ESCs.

### 3.3. MPP8 Deletion Impairs the Differentiation of ESCs

**Figure 3:**
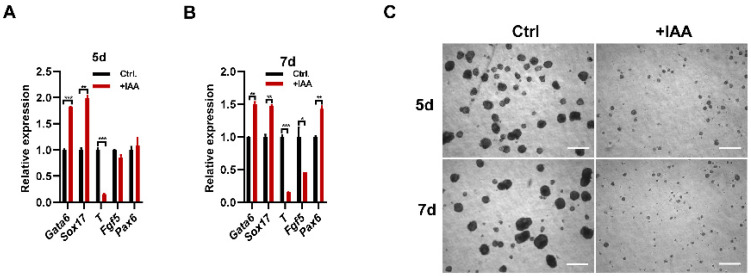
MPP8 deletion severely hampers the differentiation capacity of ESCs. (**A**,**B**) qPCR analysis reveals a notable downregulation of lineage marker genes expression in MPP8-deleted EBs compared to WT EBs at day 5 (**A**) and day 7 (**B**) (* *p* < 0.05, ** *p* < 0.01, *** *p* < 0.001). (**C**) Morphological examination demonstrates the impact of MPP8 depletion on EBs, evident in the altered appearance at day 5 and day 7. The scale bar represents 500 μm.

GO analysis revealed the association with the regulation of neurogenesis, blood vessel development, and signaling pathways such as BMP4 and TGF-β et al. (Figure 2A), suggesting the roles of Mpp8 on ESC differentiation. To investigate the effects of MPP8 deletion on the differentiation of mouse ESCs, we conducted EB differentiation assays and examined the transcription levels of well-established lineage markers. qPCR results demonstrated a significant decrease in the expression of T, a typical mesoderm marker gene in both MPP8-depleted day 5 and day 7 EBs, while the expression of endoderm marker genes Gata6 and Sox17 slightly increased (Figure 3A,B). The ectoderm marker genes Fgf5 decreased, but the neural marker gene Pax6 increased in 7D EBs (Figure 3B). This suggests that the absence of MPP8 impaired the differentiation of ESCs. Consistently, the size of both day 5 and day 7 EBs induced from MPP8-depleted ESCs was significantly smaller, compared with their WT controls (Figure 3C). In conclusion, MPP8 degradation impaired the differentiation of ESCs.

### 3.4. MPP8 Modulates Gene Expression by Influencing Epigenetic Modifications within the Promoter Region

**Figure 4:**
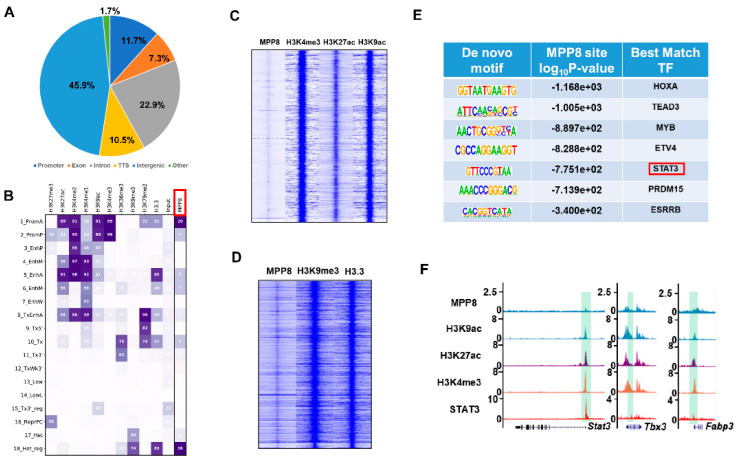
MPP8-mediated regulation of gene expression through promoter activity. (**A**) Distribution of MPP8 binding sites across the genome of mESCs shown in pie charts. TSS: transcription start site. (**B**) Chromatin states defined by ChromHMM, with horizontal columns representing chromatin states and vertical columns indicating enrichment levels of specific histone modifications. PromA (active promoer), PromP (poised promoter), EnhP (poised enhancer), EnhM1 (moderately acetylated enhancer type 1), EnhA (acetylated enhancer), EnHM2 (moderately acetylated enhancer type 2), EnhW (weakly acetylated enhancer), TxEnhA (transcribed acetylated enhancer), Tx (strong transcription), Tx5′(transcribed 5′ preferential), Tx3′ (transcribed 3′ preferential), TxWk3 (weakly transcribed 3′ preferential), Tx3′_reg(transcribed and regulatory 3′ preferential,), ReprPC (polycomb), Low (low state), LowL (lower low state), Het (heterochromatin), Het_reg (heterochromatin with regulatory activity). The last column represents MPP8 enrichment in each chromatin state. (**C**) Heatmap illustrating the enrichment of MPP8 and histone modifications H3K4me3, H3K27ac, and H3K9ac across the genome. (**D**) Heatmap analysis displaying the enrichment of MPP8, H3K9me3, and H3.3 modifications across the genome. (**E**) Motif analysis revealing enriched motifs for MPP8 binding sites. (**F**) Genome browser view displaying ChIP-seq tracks of MPP8, H3K9ac, H3K27ac, H3K4me3, and STAT3 binding at the *Stat3*, *Tbx3*, and *Fabp3* loci. Promoter regions of the indicated genes are highlighted in green.

To elucidate the mechanism underlying the regulation of pluripotency genes, such as Nanog and LIF/STAT3 target genes, by MPP8, we conducted a comprehensive bioinformatics analysis to identify MPP8 binding sites across the genome [12,29]. Our analysis revealed that a significant proportion (45.9%) of MPP8 binding sites were localized within gene promoter regions (Figure 4A). Subsequently, we examined the epigenetic landscape of these binding sites and categorized them into 18 distinct categories based on specific epigenetic modifications [44]. Notably, MPP8 exhibited prominent enrichment in the first category, which corresponds to active promoter regions characterized by histone modifications such as H3K4me3, H3K27ac, and H3K9ac (Figure 4B,C). These findings suggest that MPP8 exerts its regulatory function by interacting with and modulating the activity of promoter regions.

Interestingly, our analysis also revealed that MPP8 binds to heterochromatin regions characterized by enrichment of the H3K9me3 histone modification (Figure 4B,D), indicating a potential role of MPP8 in regulating gene expression in heterochromatin regions.

Furthermore, to explore potential synergistic effects between MPP8 and pluripotency transcription factors (TFs) in ES cells, we performed motif analysis using MPP8 ChIP-seq data. Intriguingly, we found significant enrichment of motifs corresponding to TEAD3, STAT3, and ESRRB (Figure 4E), suggesting that MPP8 binds to sites occupied by core pluripotency TFs and the effect protein of the Hippo and LIF/STAT3 pathways. Considering the regulatory effect of MPP8 on STAT3 target genes (Figure 2C–E), it is plausible that MPP8 collaboratively regulates the expression of LIF/STAT3 target genes with STAT3. Supporting this notion, ChIP-seq analysis of MPP8, H3K9ac, H3K4me3, H3K27ac, and STAT3 demonstrated co-binding of MPP8 and STAT3 at the active promoters of LIF/STAT3 target genes, such as Stat3, Tbx3, and Fabp3 (Figure 4F).

## 4. Discussion

In this study, we unveiled the pivotal role of MPP8, a member of the HUSH complex, in the maintenance and differentiation of mESCs. Notably, we observed that the inactivation of Mpp8 had a significant impact on the LIF/STAT3 signaling pathway. Furthermore, the loss of MPP8 led to the upregulation of Nanog expression. Through bioinformatics analysis, we identified that MPP8 predominantly binds to active promoter regions enriched with H3K4me3 and H3K9ac. Based on these findings, we propose that MPP8 binds to Nanog and STAT3 target genes to regulate their expression, thereby regulating the maintenance and differentiation of ESCs. 

LIF/STAT3 is essential for the maintenance of mESCs [45]. LIF binds to its receptor, the LIF receptor (LIFR), which then associates with glycoprotein 130 (gp130) to form heterodimers. This complex activates the STAT3 signaling pathway [45]. The activated form of STAT3, phosphorylated STAT3 (p-STAT3), translocates to the nucleus and induces the expression of target genes, thereby sustaining the self-renewal of mouse ESCs [37,46]. It has been demonstrated that p-STAT3 can maintain the self-renewal of mouse ESCs even in the absence of LIF, whereas the loss of *Stat3* leads to ESC differentiation [38,46]. Therefore, the *Stat3* gene is critical for the maintenance of self-renewal and pluripotency of in mouse ESCs. In this study, we observed that the deletion of MPP8 resulted in the upregulation of *Stat3* and its target genes (Figure 2D–F), indicating a potential involvement of the HUSH complex in the regulation of self-renewal and pluripotency through the LIF/STAT3 pathway. Based on our bioinformatic analysis (Figure 4), we hypothesized that MPP8 might control the expression of *Stat3* and its target genes by modulating their promoter activities. Further investigation is needed to understand how the HUSH complex regulates the activity of the LIF/STAT3 pathway.

Nanog plays crucial roles in the self-renewal and pluripotency of mouse ESCs [35,36]. Overexpression of Nanog can sustain the self-renewal of ESCs even in the absence of LIF [35,36]. Chambers et al. found that overexpression of Nanog impedes the differentiation of mESCs [35]. Thus, precise regulation of Nanog expression is vital in deciding the cell fate of ESCs. Previous studies have identified Oct4 and Sox2 as regulators of Nanog expression by binding to its promoter regions [47]. In our study, we found that MPP8 is also involved in the regulation of Nanog expression. Depletion of MPP8 leads to the upregulation of Nanog expression (Figure 1F,G and Figure 2D). Furthermore, we observed that MPP8 knockout inhibits the differentiation potential of ESCs in the absence of LIF (Figure 2G), which may be attributed to the upregulation of Nanog expression upon MPP8 degradation. It would be interesting to study the collaborative regulation of MPP8 with OCT4 and SOX2 on Nanog expression. In contrast to the inhibition of Mpp8 on Nanog expression in this study (Figure 1F,G), a minor decrease in Nanog expression was observed in the absence of MPP8 in the differentiating mESCs [31]. Considering the minor increased Nanog expression and LIF/STAT3 target genes, we generated and employed ex-Mpp8:Mpp8AID ESCs to eliminate the effect resulting from the basal degradation of the OsTIR1 system and clonal variation. Thus, the different culture conditions and the possible basic degradation from the OsTIR1 system may result in the discrepancy between the two studies [31]. Nanog prevents the endoderm differentiation of ESCs [35]. The degradation of MPP8 increased the expression of endoderm marker genes (Figure 3A,B), which might be partially explained by the downregulation of Nanog upon MPP8 degradation in differentiation conditions [31].

In our study, we found that the absence of MPP8 impaired the differentiation potential of ESCs, as manifested by the severe downregulation of T gene expression (Figure 3A,B), a typical mesoderm marker gene. Fam208a is another specific subunit of the HUSH complex [15]. The embryos lacking Fam208a could not form a normal gastrula, which showed defective mesoderm differentiation [48]. Compared with the control group, the expression of the T gene was only detected in the posterior of the Fam208a mutant embryos [48]. Interestingly, Murata et al. reported that the knockdown of Mpp8 led to an increase in T gene expression in ESCs under monolayer differentiation conditions [49]. The discrepancy of the results in the studies might be due to the distinct differentiation conditions employed and the different methods used to downregulate the expression of HUSH complex subunits. The distinct functions of different subunits of chromatin remodeling complexes in maintenance and differentiation have been reported [12,50]. Therefore, the distinct functions of Mpp8 and Fam208a in ESCs cannot be excluded, and they require further study in the future.

## Supplementary Materials

The following supporting information can be downloaded at: https://www.mdpi.com/article/10.3390/cells12162023/s1; Table S1: The list of DEG upon Mpp8 deletion in mESCs by RNA-seq analysis; Table S2: Primer sequences.
