# Supplementary material for: MPP8 Governs the Activity of the LIF/STAT3 Pathway and Plays a Crucial Role in the Differentiation of Mouse Embryonic Stem Cells
Source: Cells. 2023 Aug 8;12(16):2023. doi: 10.3390/cells12162023 (PMC10453500; doi:10.3390/cells12162023)
Supplement: Supplementary file 1 [file cells-12-02023-s001.zip › Table S2.pdf]

Table S2. Primer sequences

**Primers for qPCR**		
Targeted gene	Forward Primer (5'-3')	Reverse Primer (5'-3')
Gapdh	AGAACATCATCCCTGCATCC	CACATTGGGGGTAGGAACAC
Oct4	GTGGAGGAAGCCGACAACAATGA	CAAGCTGATTGGCGATGTGAG
Sox2	CAGGAGAACCCCAAGATGCACAA	AATCCGGGTGCTCCTTCATGTG
Nanog	TGGTCCCCACAGTTTGCCTAGTTC	CAGGTCTTCAGAGGAAGGGCGA
Tbx3	CCACCCGTTCCTCAATTTGAACAG	CGGAAGCCATTGATGGTAAAGCTG
Klf2	CCAAGAGCTCGCACCTAAAG	GTGGCACTGAAAGGGTCTGT
Klf4	GTGCAGCTTGCAGCAGTAAC	AGCGAGTTGGAAAGGATAAAGTC
Klf5	TTTCCCCCGTCACCACCAA	CTTCTGTTGTATCTGACAGG
Mpp8	GCTACCATCACCTGTGTTTGC	TGGCTCTTTTCTGGCTTTTTC
Esrrb	CAGGCAAGGATGACAGACG	GAGACAGCACGAAGGACTGC
Stat3	CTACGGGGGAGCAATGGATG	AGTTTAGTATAGTAAATCCG
Gib3	GAATCAAGGCCAGGTCTGAG	ATGCCGTGGAGTACTGGTTC
Fabp3	GCAAACTCATCCATGTGCAG	TCAGAGGGGAAAACCATGAG
Ly6g6e	TGTTACACCTGCAGCTTTGC	TGCATAGGTCCTGCTCACAG
Lama1	GATCCCATTTATGTGGGTGG	CTTGTAGGTCAAAGGCTCGG
Ppap2b	GCCTTCTACACGGGATTGTC	TGTTCCTGTCGATGATGTCC
Gata6	TTGCTCCGGTAACAGCAGTG	GTGGTCGCTTGTGTAGAAGGA
Sox17	CGCACGGAATTCGAACAGTA	GTCAAATGTCGGGGTAGTTG
T	GAGTCAAGACTCCTGGAAGGTG	CCACTCGCAGTTCGCGTTC
Fgf5	TGTGTCTCAGGGGATTGTAGG	AGCTGTTTTCTTGGAATCTCTCC
Pax6	CTGAGGAACCAGAGAAGACAGG	CATGGAACCTGATGTGAAGGAGG
